# CarSite-II: an integrated classification algorithm for identifying carbonylated sites based on K-means similarity-based undersampling and synthetic minority oversampling techniques

**DOI:** 10.1186/s12859-021-04134-3

**Published:** 2021-04-26

**Authors:** Yun Zuo, Jianyuan Lin, Xiangxiang Zeng, Quan Zou, Xiangrong Liu

**Affiliations:** 1 Department of Computer Science, Xiamen University, Xiamen, 361005 China; 2 School of Information Science and Engineering, Hunan University, Changsha, 410076 China; 3 Institute of Fundamental and Frontier Sciences, University of Electronic Science and Technology of China, Chengdu, China

**Keywords:** Carbonylation, Protein post-translational modification, K-means similarity-based undersampling, The integrated classifier, Rotation forest

## Abstract

**Background:**

Carbonylation is a non-enzymatic irreversible protein post-translational modification, and refers to the side chain of amino acid residues being attacked by reactive oxygen species and finally converted into carbonyl products. Studies have shown that protein carbonylation caused by reactive oxygen species is involved in the etiology and pathophysiological processes of aging, neurodegenerative diseases, inflammation, diabetes, amyotrophic lateral sclerosis, Huntington’s disease, and tumor. Current experimental approaches used to predict carbonylation sites are expensive, time-consuming, and limited in protein processing abilities. Computational prediction of the carbonylation residue location in protein post-translational modifications enhances the functional characterization of proteins.

**Results:**

In this study, an integrated classifier algorithm, CarSite-II, was developed to identify K, P, R, and T carbonylated sites. The resampling method K-means similarity-based undersampling and the synthetic minority oversampling technique (SMOTE-KSU) were incorporated to balance the proportions of K, P, R, and T carbonylated training samples. Next, the integrated classifier system Rotation Forest uses “support vector machine” subclassifications to divide three types of feature spaces into several subsets. CarSite-II gained Matthew’s correlation coefficient (MCC) values of 0.2287/0.3125/0.2787/0.2814, False Positive rate values of 0.2628/0.1084/0.1383/0.1313, False Negative rate values of 0.2252/0.0205/0.0976/0.0608 for K/P/R/T carbonylation sites by tenfold cross-validation, respectively. On our independent test dataset, CarSite-II yield MCC values of 0.6358/0.2910/0.4629/0.3685, False Positive rate values of 0.0165/0.0203/0.0188/0.0094, False Negative rate values of 0.1026/0.1875/0.2037/0.3333 for K/P/R/T carbonylation sites. The results show that CarSite-II achieves remarkably better performance than all currently available prediction tools.

**Conclusion:**

The related results revealed that CarSite-II achieved better performance than the currently available five programs, and revealed the usefulness of the SMOTE-KSU resampling approach and integration algorithm. For the convenience of experimental scientists, the web tool of CarSite-II is available in http://47.100.136.41:8081/

**Supplementary Information:**

The online version contains supplementary material available at 10.1186/s12859-021-04134-3.

## Background

Protein carbonylation is an irreversible chemical modification in oxidative stress, which refers to the side chain of amino acid residues being attacked by reactive oxygen species and finally converted into carbonyl products [1]. Modification of the protein by carbonylation will cause changes in the structure of the protein, causing it to lose its original biological function, eventually leading to cell and tissue dysfunction and pathophysiological changes in the body. The level of protein carbonylation has only been used for a long time to evaluate the degree of oxidation of biological organisms as an indicator to measure the oxidative damage of proteins. However, studies have shown that protein carbonylation caused by reactive oxygen species is involved in the etiology and pathophysiological processes of aging, apoptosis and various neurodegenerative diseases.

Under oxidative stress induced by different diseases, carbonylation has certain selectivity for proteins, that is, some proteins are easily carbonylated, while others are not easily carbonylated [1]. Taking the cytoskeleton as an example, glial fibrillary acidic protein (GFAP) is the protein most vulnerable to oxidative damage in multiple sclerosis [2], Pick's disease [3], and aging [4]. Its carbonylation level increased, however, it decreased in patients with Alzheimer's disease [4]. In addition, the β-actin carbonylation level of another cytoskeleton molecule increased in Alzheimer's disease [4] and multiple sclerosis [2], but decreased in aging.

After the carbonylated protein is produced, it cannot be repaired by the body's antioxidant defense mechanism, so it will slowly accumulate over time, resulting in the change or loss of the functions of key enzymes in various signaling pathways, and then trigger a series of diseases related to protein carbonylation: aging, neurodegenerative diseases (such as Alzheimer's disease, Parkinson's disease, and Multiple sclerosis), inflammation, diabetes, and tumor (such as Uterine fibroids, malignant prostate cancer, and breast cancer). These all indicate that protein carbonylation modification is not only a sign of the degree of cell oxidation, but also involved in the pathophysiological process of the disease.

For the following reasons, it is necessary to develop computational methods for prediction of carbonylation sites. (1) Since the carbonylation site is the decisive factor for the functional change or deletion of the carbonylated protein, the identification of the carbonylation site and its role in the protein are crucial for understanding the protein carbonylation process and related pathogenesis, and current experimental approaches used to identify carbonylation sites are expensive, time-consuming, and limited in protein processing abilities. Computational prediction of the carbonylation residue location in protein post-translational modifications enhances the functional characterization of proteins. (2) Corresponding prediction and analysis of protein carbonylation sites can give experimental researchers a pre-experimental evaluation to make them aware of the occurrence probability and corresponding number of carbonylation sites on the target protein, allowing for more targeted experiments. (3) In order to reveal the pathophysiological process of the diseases (aging, neurodegenerative diseases, inflammation, diabetes, tumor and so on), the prediction of protein carbonylation sites is significance for in-depth understanding the biological functions and developing effective drugs. Therefore, it is very important to establish an online prediction platform with clear interface and easy identification of carbonylation sites.

It is worth noting that only four types of residues are particularly sensitive to carbonylation, and they are lysine (K), proline (P), arginine (R), and threonine (T) residues [5]. In the past several years, a series of computational methods and tools have been proposed for identifying carbonylation proteins and sites [5–13]. However, the predictive performance of protein carbonylation sites is still unsatisfactory compared with other post-translational modification sites (PTMs) in proteins. Therefore, for the sake of satisfying the modern requirement to develop efficient high-throughput computing tools, supererogation is still required to move forward a single step, improving the predictive performance of carbonylation sites.

**Fig. 1:**
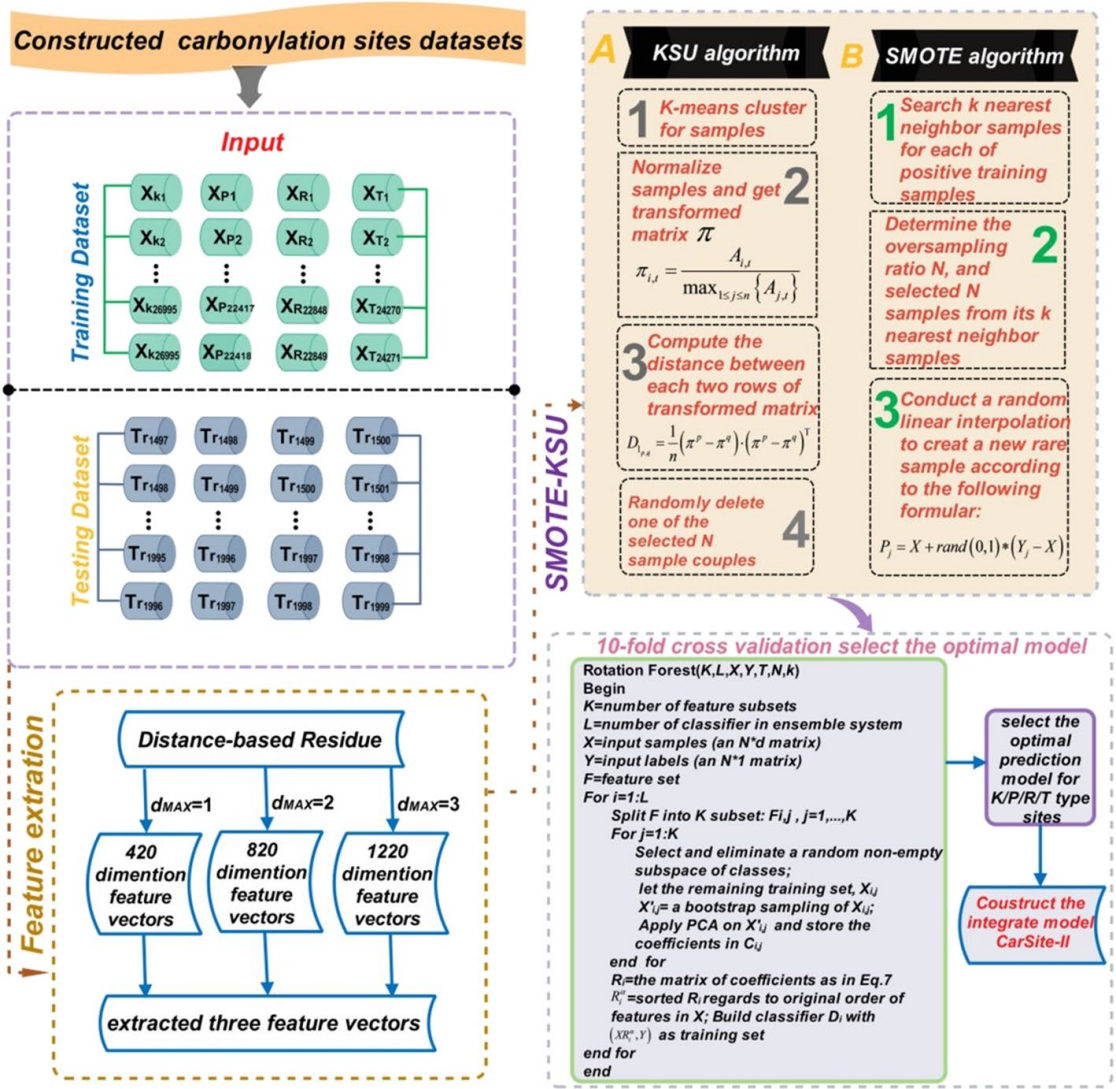
Conceptual framework of CarSite-II

In the current study, K-means similarity-based undersampling (KSU) and the synthetic minority oversampling technique (SMOTE) were introduced and combined to construct balance training datasets for K, P, R, and T carbonylation modification sites, respectively. SMOTE [14] was utilized to synthesize K, P, R, and T carbonylation sites (positive training samples) by using experimentally validated positive training samples, while KSU was applied to eliminate samples with little information that have little impact on classification and redundant samples. The resampling method combining KSU and SMOTE was conveniently named SMOTE-KSU. Based on constructing positive and negative training samples using the SMOTE-KSU resampling method, a novel computational predictive tool was developed. This tool, named as CarSite-II, was created to distinguish carbonylation sites from non-carbonylation sites through distance-based residue (DR) feature extraction strategy and Rotation Forest integrated algorithm-based “support vector machine” (SVM) subclassification. According to the related results obtained by tenfold cross-validation and independent tests, CarSite-II achieves remarkably better predictive performance than existing predictor tools. Figure 1 shows the flow chart for constructing four optimal models for K/P/R/T carbonylation sites, CarSite-II. The Fig. 1 mainly consists of the following four parts to improve the prediction accuracy of K/P/R/T carbonylation sites: (1) construct protein carbonylation training and testing dataset. (2) use the feature extraction strategy of distance-based residue to formulate K/P/R/T carbonylation samples. (3) KSU undersampling method and SMOTE oversampling technique were incorporated to balance the training dataset. (4) The tenfold cross validation was used to select the optimal model.

## Results

### Amino acid composition of carbonylation sites

**Fig. 2:**
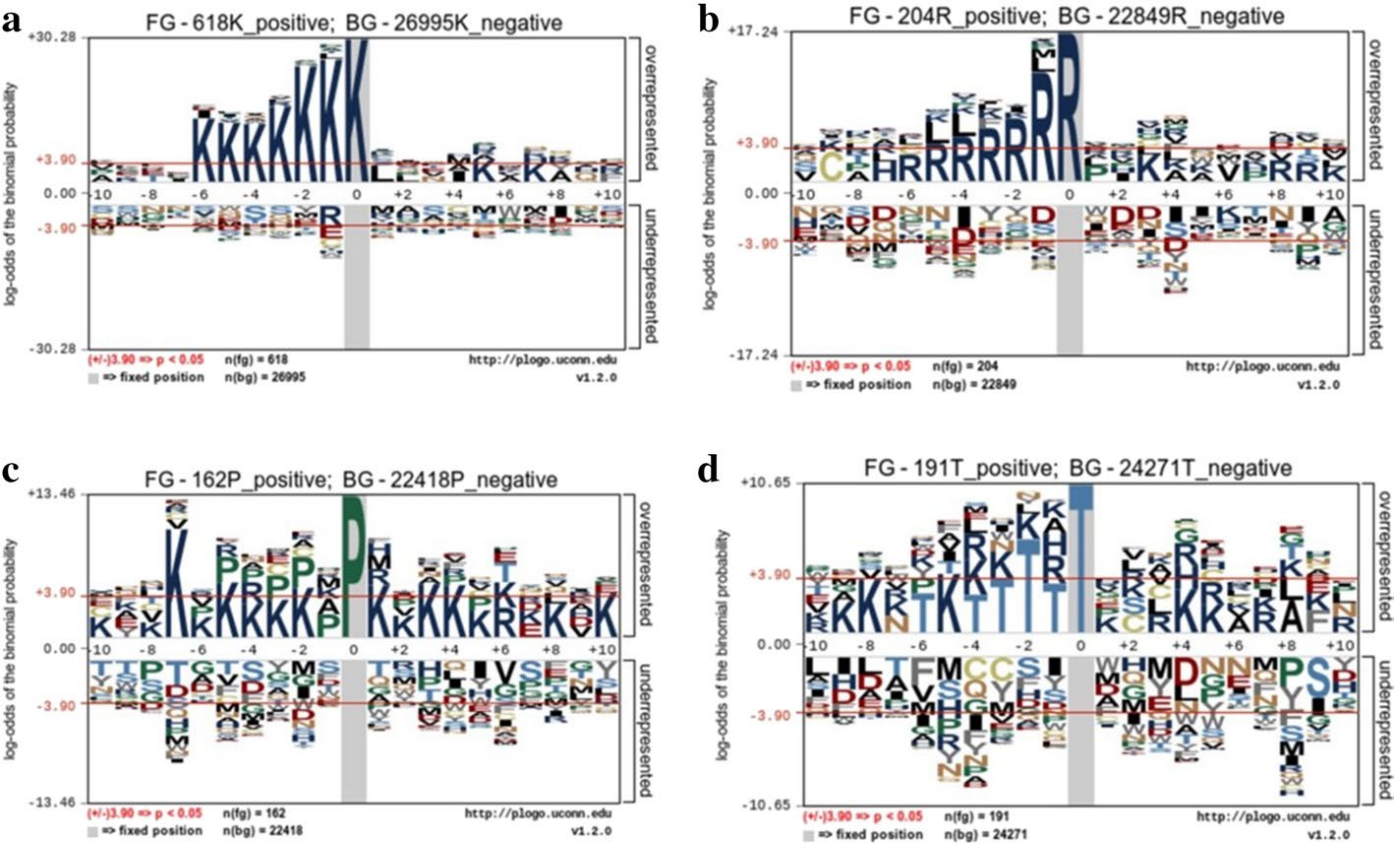
Sequence logo of four carbonylated residues in positive and negative training dataset. **a** Sequence log of Lys (K). **b** Sequence log of Arginine (R). **c** Sequence log of Proline (P). **d** Sequence log of Threoline (T)

To explore the position-specific differences in amino acid residue distributions in the carbonylation and non-carbonylation sites, training samples were submitted to the pLogo web server [15] (https://plogo.uconn.edu/), and the sequence logo of four carbonylated residues was shown in Fig. 2. As we can see from Fig. 2, Lys (K) at position − 6, − 5, − 4, − 3, − 2, and − 1 was significantly overrepresented in K carbonylation site sequence logo, Arginine (R) at position − 5, − 4, − 3, − 2, and − 1 was significantly overrepresented in R carbonylation site sequence logo, Proline (P) was not significantly overrepresented in P carbonylation site sequence logo, and Threonine (T) at position − 3 and -2 was significantly overrepresented in T carbonylation site sequence logo.

### Balance the training dataset and select optimal parameters of DR and rotation forest

As described in Material and methods, each sequence in the training dataset can be encoded by DR, and SMOTE oversampling and KSU undersampling were used to resample the training dataset to make the same size of positive and negative training samples. We calculated the number of samples (*N*) removed from the negative samples or added to the positive samples during the process of resampling according to the following formula [16]:

1 $$N = round\left[ {\left( {k_{1} \times n_{0} } \right) - \left( {k_{0} \times n_{1} } \right)} \right],$$

where $k_{0} = 0.5,k_{1} = 0.5$, and $n_{0}$ or $n_{1}$ represented the number of sequences included in the negative or positive training samples. Therefore, *N* was 13189/11128/11323/ 12040 for K/P/R/T carbonylation sites, respectively.

**Fig. 3:**
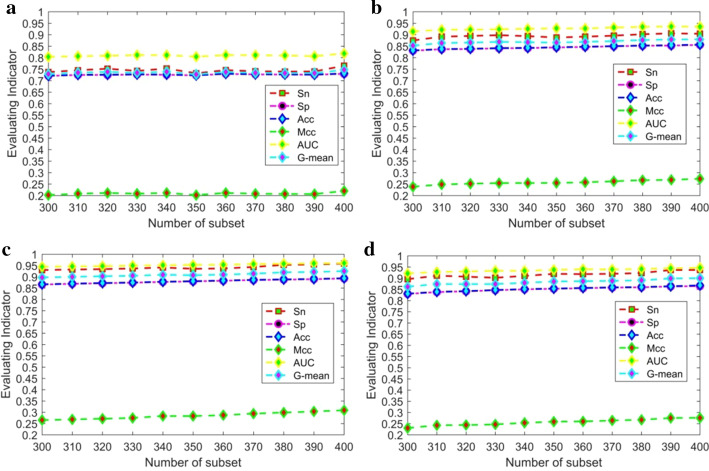
The prediction performance for K/P/R/T carbonylation sites by tenfold cross validation. **a** The prediction performance for K carbonylation sites. **b** The prediction performance for R carbonylation sites. **c** The prediction performance for P carbonylation sites. **d** The prediction performance for T carbonylation sites

SVM was used for subclassification of the Rotation Forest algorithm, and the parameters of the Rotation Forest algorithm were set to the following: *K* ranged from 300 to 400, with an interval of 10, and the number of subclassifiers was set as five. The concrete results of the K/P/R/T carbonylation sites 10-fold cross validation were listed in the Additional File 1: SupTable (SubTable1.1–SubTable1.4. The predictive performance of K/P/R/T carbonylation sites by 10-fold cross validation). As we can see from SupTable (SubTable1.1–SubTable1.4. The predictive performance of K/P/R/T carbonylation sites by 10-fold cross validation), while $d_{MAX} = 3,K = 400$, the K carbonylation dataset can get the best prediction results. While $d_{MAX} = 2,K = 400$,$d_{MAX} = 1,K = 400$, $d_{MAX} = 3,K = 400$, the P/R/T carbonylation dataset can get the best prediction results, respectively. To improve the predictive performance of carbonylation sites, the parameters selected above were used to construct the final integrated prediction model for K/P/R/T carbonylation sites. The prediction performance for K/P/R/T carbonylation sites based on the Rotation Forest integrated algorithm by tenfold cross-validation is shown in Fig. 3.

As we can see from Fig. 3, while the number of the subsets in the Rotation Forest integrated algorithm was 400 (K/P/R/T), Sn, Sp, Acc, and MCC all reached the best values. In Fig. 3, the Sn, Sp, Acc, MCC, AUC, and G-mean indicated the Sn, Sp, Acc, MCC, AUC, and G-mean average values of three features (DR(1), DR(2), DR(3)) based on the selected the number of subsets, respectively. For example, when the number of subset K = 300, the evaluating indicators Sn = (0.7520 + 0.7228 + 0.7375)/3 = 0.7374,Sp = (0.7268 + 0.7124 + 0.7228)/3 = 0.7207,Acc = (0.7279 + 0.7128 + 0.7233)/3 = 0.7213, MCC = (0.2129 + 0.1906 + 0.2038)/3 = 0.2024,AUC = (0.8150 + 0.7963 + 0.8002)/3 = 0.8038,andG-mean = (0.7393 + 0.7176 + 0.7301)/3 = 0.7290.

### The effectiveness of resampling approach

The related predictive results of the independent tests were utilized to clarify the effectiveness of our combination of the SMOTE-KSU resampling method. The comparison results are listed in Table 1 for without resampling, conducting SMOTE only for positive sequences, conducting KSU only for negative sequences, and conducting SMOTE-KSU resampling for the training dataset.

**Table 1:** Comparison of different resampling methods on our independent test data

Resample method	Sn (%)	Sp (%)	Acc (%)	Mcc	AUC	G-mean
*K*						
Without resampling	5.13	95.16	93.77	0.0017	0.4959	0.2209
SMOTE	41.88	98.43	97.55	0.3395	0.8868	0.6420
KSU undersampling	70.94	86.54	86.30	0.2025	0.8096	0.7835
CarSite-II	89.74	98.35	98.21	0.6358	0.9603	0.9395
*P*						
Without resampling	0	100	99.70	NaN	0.6116	0
SMOTE	50.00	97.61	97.47	0.1658	0.8512	0.6986
KSU undersampling	31.25	99.64	99.44	0.2524	0.8810	0.5580
CarSite-II	81.25	97.97	97.92	0.2910	0.8768	0.8922
*R*						
Without resampling	3.70	96.65	95.81	0.0018	0.6210	0.1892
SMOTE	27.78	97.96	97.33	0.1627	0.8695	0.5216
KSU undersampling	46.30	88.18	87.81	0.0996	0.7631	0.6389
CarSite-II	79.63	98.12	97.96	0.4629	0.9236	0.8839
*T*						
Without resampling	8.33	98.43	98.10	0.0327	0.7539	0.2864
SMOTE	12.50	98.36	98.04	0.0510	0.8250	0.3506
KSU undersampling	45.83	92.29	92.11	0.0857	0.8120	0.6504
CarSite-II	66.67	99.06	98.94	0.3685	0.8602	0.8127

We discovered that CarSite-II based on the SMOTE-KSU resampling approach reached the best performance, with MCC of 0.6358/0.2910/0.4629/0.3685 for K/P/R/T carbonylation sites, respectively. Additionally, KSU undersampling achieved the second best prediction performance, with Sn values of 70.94% for K carbonylation sites. The values of Sn obtained by without resampling, and SMOTE oversampling for K/P/R/T carbonylation sites, and KSU undersampling for P/R/T carbonylation sites, were less than 50%. The major reason for this may be imbalance of training dataset. The ratios between training positive samples and training negative samples for K carbonylation sites were over 1:22 (618:13807), 1:43 (618:26995), and 1:1.9 (13807:26995) corresponding to KSU undersampling, without resampling, and SMOTE oversampling. The ratios between training positive samples and training negative samples for P/R/T carbonylation sites were also very different (i.e. the training dataset is extremely unbalanced) for KSU undersampling, without resampling, and SMOTE oversampling. Thus, we did not consider them further.

**Fig. 4:**
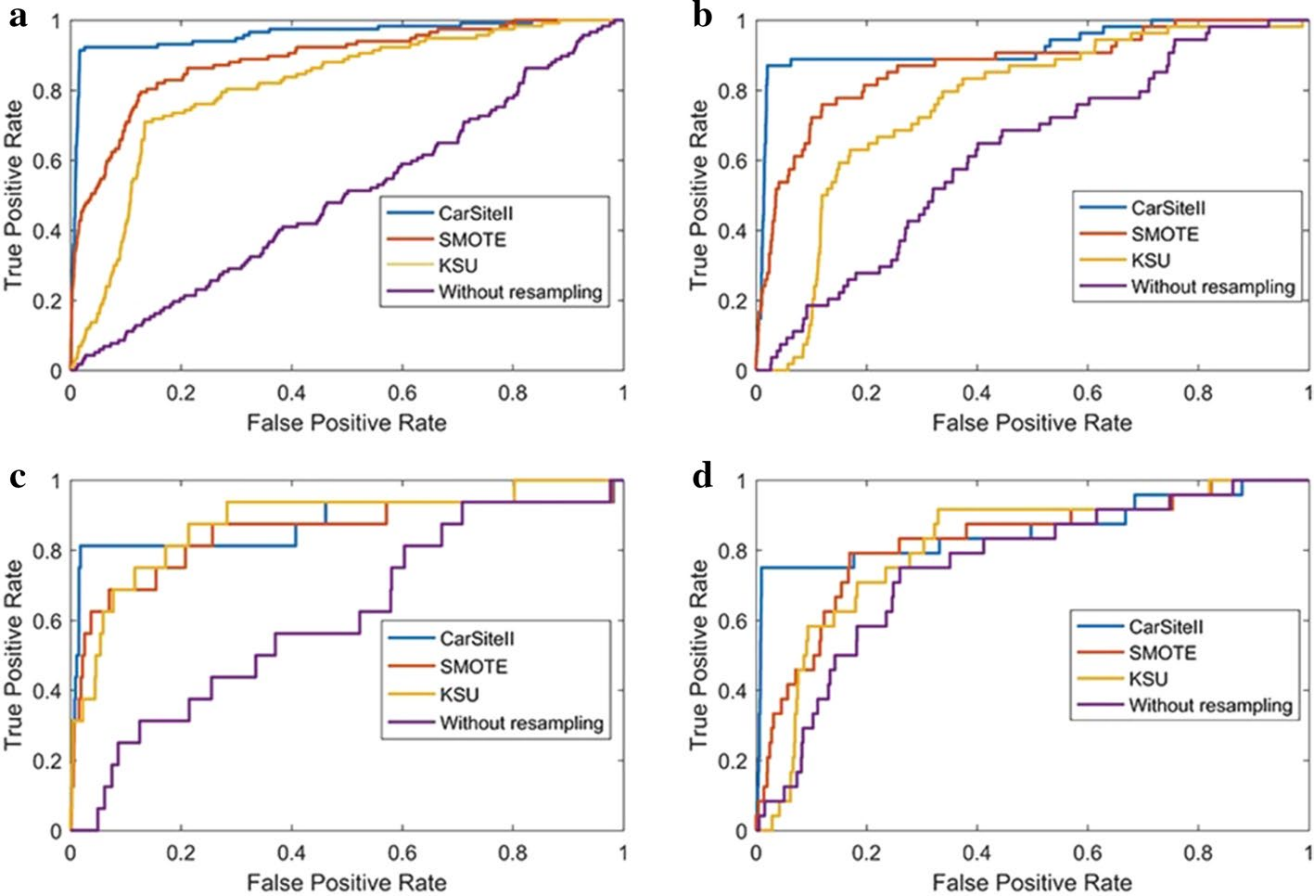
The ROC curves comparison of different resampling methods K/P/R/T carbonylation sites on our independent test dataset. **a** Comparison of different resampling methods for K carbonylation datatset. **b** Comparison of different resampling methods for R carbonylation datatset. **c** Comparison of different resampling methods for P carbonylation datatset. **d** Comparison of different resampling methods for T carbonylation datatset

In order to further look at comparative performance, the ROC curves comparision of different resampling methods for K/P/R/T carbonylation sites on our independent test dataset was given in Fig. 4.

### Comparison with other prediction methods and discuss

To better test and verify the performance of CarSite-II, we compared CarSite-II with three currently available programs in our independent test. The first predictive tool, CarSPred, based on four types of features and mRMR feature selection agorithm with weighted support vector machine [7]. In 2016, Lv et al. based three types of features and IFS feature selection algorithm with weighted support vector machine [7] to construct the predictive tool CarSPred.Y [9]. In our previous work, the one-sided selection undersampling algorithm was used to balanced training dataset, and hybrid combination of four feature extraction strategies with support vector machine to build the tool, CarSite [13].

In terms of the dataset used to build the above three currently available programs and the prediction threshold used for each method, CarSPred used 266K/119R/116T/114P human carbonylation sites and 1802K/754R/702T/716P human non-carbonylation sites to construct the tool, and used 34K/17/5T/12P carbonylation sites and 147K/93R/30T/76P non-carbonylation sites from the human and other mammals to construct the test dataset, and the determination threshold can be assigned to any value from 0 to 1 which is set to 0.5 by default. CarSPred.Y used 86K/56R/44T/59P carbonylation sites and 536K/363R/271T/358P non-carbonylation sites from yeast proteins to construct the training model, and the determination threshold was same with CarSPred. CarSite used the same cabonylation proteins with CarSPred and the threshold was set as 0.5. In this study, we used the threshold of 0.5 to make relevant comparisons.

CarSite-II was compared with CarSPred.Y, CarSPred, and CarSite. The relevant results to identify carbonylation sites are shown in the Table 2. We can see from Table 2 that although the value of Sp by CarSite-II was about 0.45% lower than that for CarSPred for K carbonylation sites, the values of Sn was about 85.47% higher. CarSite-II gained the best Sn of 89.74%, 81.25%, 79.63% and 66.67% for K/P/R/T carbonylation sites, respectively, which generally lead to 18.8%, 12.5%, 24.07% and 8.34%, and 58.97%, 25%, 53.7% and 33.34% improvement with regard to the second and third best classifiers, respectively. In terms of another evaluation criterion, MCC, CarSite-II gained the best MCC of 0.6358, 0.2910, 0.4629, and 0.3685 for K, P, R, and T carbonylation sites, respectively. CarSite gained the second best MCC of 0.2203, 0.0785, 0.0806 and 0.0840 for K, P, R, and T carbonylation sites, respectively. CarSPred.Y gained the third best MCC of 0.1083, 0.0773, 0.0735 and 0.0765.

**Table 2:** Comparison of CarSite-II with other all available predictors on our independent test set

Classifier	TP	FP	Sn (%)	Sp (%)	Acc (%)	Mcc	AUC	G-mean
*K*								
CarSPred.Y	36	547	30.77	92.65	91.69	0.1083	–	0.5339
CarSPred	5	89	4.27	98.80	97.34	0.0343	–	0.2055
CarSite	83	870	70.94	88.30	88.04	0.2203	0.8897	0.7915
CarSite-II	105	123	89.74	98.35	98.21	0.6358	0.9603	0.9395
*P*								
CarSPred.Y	11	868	68.75	83.68	83.63	0.0773	–	0.7585
CarSPred	3	315	18.75	94.08	93.85	0.0296	–	0.4200
CarSite	9	587	56.25	88.96	88.86	0.0785	0.8671	0.7074
CarSite-II	13	108	81.25	97.97	97.92	0.2910	0.8768	0.8922
*R*								
CarSPred.Y	14	387	25.93	93.51	92.91	0.0735	–	0.4924
CarSPred	10	321	18.52	94.62	93.94	0.0543	–	0.4186
CarSite	30	1236	55.56	79.28	79.07	0.0806	0.7715	0.6637
CarSite-II	43	112	79.63	98.12	97.96	0.4629	0.9236	0.8839
*T*								
CarSPred.Y	8	335	33.33	94.85	94.63	0.0765	–	0.5623
CarSPred	4	271	16.67	95.84	95.54	0.0377	–	0.3997
CarSite	14	805	58.33	87.63	87.52	0.0840	0.7998	0.7150
CarSite-II	16	61	66.67	99.06	98.94	0.3685	0.8602	0.8127

Furthermore, since the original training datasets were friendly offered by PTMPred [6], CarSpred [7], iCar-PseCp [8] and CarSite [13], CarSite-II was compared with these methods using tenfold cross-validation according to the results listed in their works. As shown in Table 3, CarSite-II was significantly better than PTMPred, CarSpred, iCar-PseCp and CarSite.

**Table 3:** A comparisons of CarSite-II with other methods based on the tenfold cross validation on the same 250 carbonylated proteins

Predictor	Sn (%)	Sp (%)	Acc (%)	MCC	AUC	G-mean
*K*						
PTMPred	23.45	92.99	88.59	0.1892	0.6858	0.4670
CarSpred	23.17	92.43	87.22	0.2268	0.6849	0.4628
iCar-PseCp	45.18	99.25	84.43	0.5906	0.8728	0.6696
CarSite	66.33	73.40	72.45	0.2936	0.7250	0.6978
CarSite-II	85.19	81.93	82.73	0.6074	0.8739	0.8354
*P*						
PTMPred	21.43	93.20	82.93	0.2573	0.6903	0.4469
CarSpred	25.34	93.28	82.93	0.2331	0.7163	0.4862
iCar-PseCp	48.20	98.54	86.79	0.6006	0.8484	0.6892
CarSite	70.58	73.67	73.26	0.3280	0.7337	0.7211
CarSite-II	92.31	80.88	82.72	0.5816	0.8433	0.8641
*R*						
PTMPred	20.02	90.99	86.64	0.1878	0.5981	0.4268
CarSpred	25.47	93.39	86.22	0.2245	0.7158	0.4877
iCar-PseCp	46.67	99.57	84.23	0.6076	0.8668	0.6817
CarSite	65.50	65.95	65.88	0.2252	0.6295	0.6572
CarSite-II	90.00	82.35	83.16	0.5110	0.8741	0.8609
*T*						
PTMPred	22.38	91.36	88.39	0.2186	0.6563	0.4522
CarSpred	21.39	93.42	86.61	0.2040	0.7134	0.4470
iCar-PseCp	50.68	98.58	86.17	0.6185	0.8603	0.7068
CarSite	68.33	73.56	72.82	0.3226	0.7314	0.7090
CarSite-II	99.91	82.86	85.37	0.6437	0.9214	0.9099

Meanwhile, we used Wilcoxon signed rank test to verify the significant of different methods in Table 1 and Table 2. The relevant results are listed in Additional File 2: SubTable 2. The Wilcoxon signde rank of the K/P/R/T carbonylation sites. Two-sided test for the null hypothesis that x–y comes from a distribution with zero median at the 5% significance level. As we can see from the Additional File 2: SubTable 2. The Wilcoxon signde rank of the K/P/R/T carbonylation sites, the values of H are all 1. In other words, it indicates a rejection of the null hypothesis at the 5% significance level.

These results indicated that CarSite-II is a significant improvement over all currently available tools.

## Discussion

Protein carbonylation is a type of protein oxidative damage, which is itself an irreversible chemical modification in oxidative stress, which refers to the side chain of amino acid residues being attacked by reactive oxygen species and finally converted into carbonyl products [1]. Modification of the protein by carbonylation will cause changes in the structure of the protein, causing it to lose its original biological function, eventually leading to cell and tissue dysfunction and pathophysiological changes in the body. The study by Nabeshi and his team showed that carbonyl modification of purified Cu, Zn-SOD increased by the reaction with H_2_O_2_. Therefore, progressive accumulation of oxidative damage to Cu, Zn-SOD, may cause dysfunction of defense systems against oxidative stress in SAMP8 with a higher oxidative states, leading to acceleration of aging. Furthermore, carbonyl modification of HCNP-pp may be involved in pathophysiological alterations associated with deterioration in the learning and memory in the brain seen in SAMP8 [17].

## Conclusions

In the current study, a novel resampling approach, SMOTE-KSU, was proposed to balance the size of small and large samples. A balanced dataset based on SMOTE-KSU resampling, the optimal parameters of DR, and Rotation Forest for K, P, R, and T carbonylation sites were selected according to the related results of tenfold cross-validation, respectively. Hereafter, we applied a majority voting strategy to develop the integrated predictor CarSite-II based on the Rotation Forest integrated algorithm. The related results revealed that CarSite-II achieved better performance than the currently available five programs, and revealed the usefulness of the SMOTE-KSU resampling approach and integration algorithm. Since Deep learning plays an important supplementary role in sequence analysis, we may construct a Deep learning predict model to better identify carbonylation sites in the future work. Our future work aims at extending this work to other bioinformatics sequence recognition. For the convenience of experimental scientists, we have given a web-server guide on how to use the CarSite-II web tool to get their desired results without the need to follow the complicated mathematic equations that presented just for the integrity in developing the web tool CarSite-II. The detailed steps are shown in the Additional file 3: SubTable 3. Web-Server Guide.

## Material and methods

### Data collection and pre-processing

The dataset gathered from CarbonylDB [18], which was the only existing database or resource for carbonylated proteins or sites, was used in the current study. From CarbonylDB, we collected 685, 178, 211, and 208 experimentally verified K, P, R, and T carbonylated sites on 468 human proteins as positive samples, while the remaining 42523K, 35302P, 33050R, and 34774T carbonylated sites on the same 468 human proteins were regarded as negative samples to construct the training dataset. Meanwhile, CD-HIT [19] was utilized as the software for the removal of redundant samples. For a cut-off of 40% identity, 445 carbonylated human proteins were retained. Subsequently, for a cut-off of 70% identity, some carbonylated sites with a high identity of the 445 carbonylated proteins were removed. Finally, a total of 618K, 162P, 204R, and 191T carbonylated sites (the positive training samples) and 26995K, 22418P, 22849R, and 24271T non-carbonylated sites (the negative training samples) were collected.

Furthermore, to avoid overestimating the predictive performance resulting from overfitting of the training dataset and to evaluate the proposed model's real predictive performance, an independent testing set was constructed. The independent testing set was constructed by collecting the proteins of rats, yeast, and mice from CarbonylDB [18] (298 rat proteins, 239 yeast proteins, and 90 mouse proteins), and CD-HIT [19] was used to remove redundant proteins and samples. For a cut-off of 40% identity, 277 rat proteins, 222 yeast proteins, and 76 mouse proteins were retained. Subsequently, cd-hit-2d [19] was used to control for homology between training and test datasets and within the test dataset. For a cut-off of 40% identity, 223 rat proteins, 209 yeast proteins, and 42 mouse proteins were retained. Then, for a cut-off of 70% identity, some carbonylated sites with a high identity of the retained three species of carbonylated proteins were removed, a total of 117K, 16P, 54R, and 24T carbonylated sites were collected. For collecting negative test samples, after having filtered out fragments with 30% identity, the final negative test dataset comprised 7439K, 5318P, 5966R, and 6507T non-carbonylated sites. Finally, the independent test set contained 117 K, 16P, 54R, and 24T carbonylated sites and 7439K, 5318P, 5966R, and 6507T non-carbonylated sites. Table 4 shows the concrete statistics of the training dataset and independent test dataset.

**Table 4:** Summary of K/P/R/T carbonylation samples and non- carbonylation samples

Dataset	Subset	Carbonylation type and number of samples			
		K	P	R	T
Training dataset	Positive	618	162	204	191
	Negative	26,995	22,418	22,849	24,271
Independent test dataset	Positive	117	16	54	24
	Negative	7439	5318	5966	6507

### Distance-based residue features extraction strategy

DR, proposed by Liu et al. [20], was used to convert carbonylation and non-carbonylation protein sequences into valid numerical vectors in this study. Given a protein sequence *R* with L amino acid residues, i.e.

2 $$R = R_{1} R_{2} \ldots R_{i} \ldots R_{L - 1} R_{L}$$

where $R_{i}$ represents the *i*th position amino acid residue along a given protein sequence. The DR measure of *R* can be defined as:

3 $$F_{{d_{MAX} }} \left( R \right) = \left[ {D_{0} \left( R \right),D_{1} \left( R \right), \ldots ,D_{k} \left( R \right), \ldots ,D_{{d_{MAX} }} \left( R \right)} \right]$$

The dimension of $F_{{d_{MAX} }} \left( R \right)$ is $20 + 20 \times 20 \times d_{MAX}$, where 20 indicated 20 kinds of naïve amino acid residues:

4 $$D_{k} \left( R \right) = \left\{ {_{{\left[ {T_{AA}^{k} \left( R \right),T_{AC}^{k} \left( R \right), \ldots ,T_{YY}^{k} \left( R \right)} \right]\left( {1 \le k \le d_{MAX} } \right)}}^{{\left[ {T_{A}^{0} \left( R \right),T_{C}^{0} \left( R \right), \ldots ,T_{Y}^{0} \left( R \right)} \right]\left( {k = 0} \right)}} } \right.$$

$i \in \left\{ {A,C,D,E,F,G,H,I,K,L,M,N,P,Q,R,S,T,V,W,Y} \right\}$, $T_{i}^{0} \left( R \right)$ was the occurrences of the amino acid residue *i*, and $T_{ij}^{d} \left( R \right)$ was the occurrences of the amino acid residue pair (*i*, *j*). $d_{MAX}$ represented the maximum distance between amino acid residue pair (*i*, *j*), and in this study, we set it as 1, 2, and 3, respectively.

**Fig. 5:**
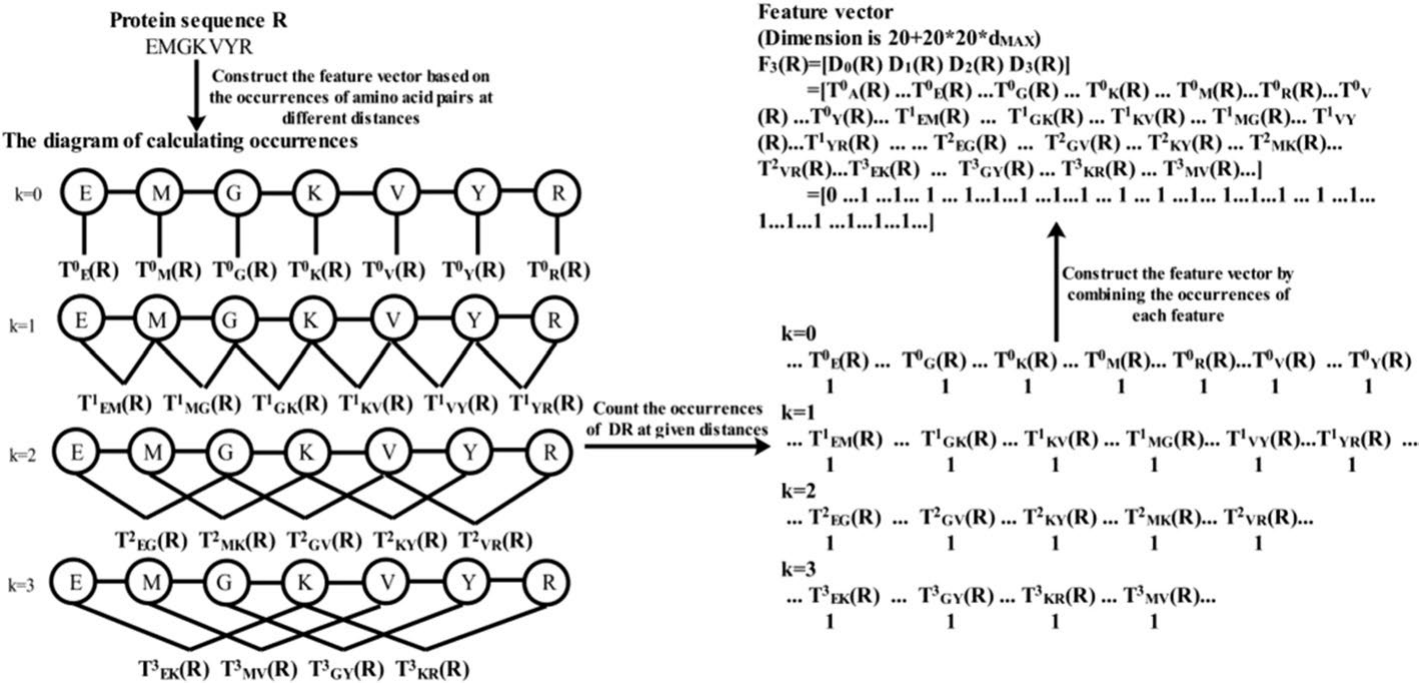
The process of generating DR feature vectors

In order to make researchers further understand the concrete process of converting a carbonylation or non-carbonylation protein sequence into valid numerical vector, the concrete process of generating DR feature vectors shown in Fig. 5.

### Resampling methods

#### The synthetic minority oversampling technique

The SMOTE algorithm is the most frequently and commonly used oversampling method [21–23]. The primary idea of the SMOTE algorithm is to place synthetic example along the line segments connecting existing rare examples [14]. We briefly present the following:

Given a positive training sample $X$, and searching for its $k$ nearest neighbor examples (usually set as 5), assume that the oversampling ratio was $N$, then $N$ samples were selected from its $k$ nearest neighbor examples. Conduct a random linear interpolation between $X$ and $Y_{j} \left( {j = 1,2, \ldots ,N} \right)$ to create a new rare sample $P_{j}$ according to the formula (5):

5 $$P_{j} = X + rand\left( {0,1} \right) * \left( {Y_{j} - X} \right),\quad j = 1,2, \ldots ,N.$$

where $rand\left( {0,1} \right)$ represents the random number generated in the interval $\left( {0,1} \right)$. For a concrete explanation of the SMOTE algorithm, please refer to References [14].

#### Kmeans similarity-based undersampling

KSU is proposed in this study based on a novel methodology that is capable of removing redundant samples [16, 24]. The main idea of KSU is to find out the similarity between the negative training samples. The detailed steps are as following:

1. First, make the convergence of negative training samples into *k* clusters using a K-means clustering algorithm (we set as *k* = 6 in this study).
2. Then, for each cluster of negative training samples, suppose that $A \in {\mathbb{R}}^{n \times d}$ indicated all negative samples in the *k*-th cluster, $d$ was the dimensionality of the extracted features, and $n$ indicated the number of all negative samples in the *k*-th cluster. Normalize all columns of the matrix $A \in {\mathbb{R}}^{n \times d}$ and get a transformed matrix $\pi$, where the element at its *i*-th row and *j*-th column can be represented as:
  6 $$\pi_{i,t} = \frac{{A_{i,t} }}{{\max_{1 \le j \le n} \left\{ {A_{j,t} } \right\}}},\quad i = 1,2, \ldots ,n, \, t = 1,2, \ldots ,d$$
3. Compute the distance between every two rows of the transformed matrix $\pi$ to get a symmetrical square distance matrix $D_{1}$, where the element at its *p*-th row and *q*-th column can be represented as:
  7 $$D_{{1_{p,q} }} = \frac{1}{n}\left( {\pi^{p} - \pi^{q} } \right).\left( {\pi^{p} - \pi^{q} } \right)^{T}$$
  where $\pi^{p}$ and $\pi^{q}$ represented vectors consisting of all elements of the *p*-th and *q*-th rows of the matrix $\pi$, respectively. It was clear that the elements located on the main diagonal of the symmetrical square distance matrix $D_{1}$ are zero. Because the matrix $D_{1}$ was a symmetry matrix, only the upper triangle of the matrix $D_{1}$ was considered in the below, and $D_{1}$ was defined as a *dissimilarity matrix*.
4. The smaller the element $D_{{1_{p,q} }}$, the more “similar” the samples $A_{p}$ and $A_{q}$. The couples of samples were rearranged based on this similarity index and in *N* the most similar couples of samples, we randomly selected one of the samples to eliminate.

### Rotation forest integration algorithm

The Rotation Forest integrated algorithm was firstly proposed in 2006 by Rodriguez et al. [25]. Their goal was to develop a powerful integrated learning algorithm for noise and rotation of data. The basic idea of the Rotation Forest integrated algorithm was based on Random Forest, and we used it to consturct the integrated predictive model. The algorithm is as follows: In the dataset $X = [x_{1} , \ldots x_{n} ]^{T}$ containing *n* features, *X* is an *N* × *n* matrix with a sample size of *N*, which constitutes the feature set *F*, $Y = [y_{1} , \ldots y_{n} ]^{T}$ is the corresponding labels. There are two important parameters in the algorithm that need to be defined: the number of feature subsets *K*, and the number of classifier in ensemble system *L*. In an integrated classification system, generally includes $L = D_{1} , \ldots D_{L}$ sub-classifiers. The concrete algorithm is described as follows.

The first step, the feature set $F$ was randomly divided into *K* subsets, each of which contained *M* = *n*/*K* features. For simplicity, generally set *K* as a factor of *n*.

The second step, $F_{ij}$ is the *j*-th feature subset used to train the sub-classifier $D_{i}$. Corresponding to each feature subset $F_{ij}$, $X_{ij}$ is a subset of samples containing feature $F_{ij}$ in $X$. Using bootstrap resampling technology for $X_{ij}$, 75% of the samples are randomly and repeatedly extracted to form a new bootstrap sample set $X_{ij}^{\prime }$. Then, we performed the principal component analysis on $X_{ij}^{\prime }$, and recorded the generated coefficient matrix $C_{ij} = [a_{ij}^{(1)} , \ldots a_{ij}^{{(M_{k} )}} ]$. It is worth noting that the possible eigenvalue is zero, resulting in $M_{j} \le M$. The purpose of a linear transformation on feature subsets rather than full data sets is to avoid constructing subclassifiers with the same coefficient matrix.

The third step, construct a sparse “rotation” matrix $R_{i}$ with the obtained coefficient matrix $C_{ij}$:

8 $$R_{i} = \left[ {\begin{array}{*{20}l} {C_{i1} } \hfill & 0 \hfill & \cdots \hfill & 0 \hfill \\ 0 \hfill & {C_{i2} } \hfill & \cdots \hfill & 0 \hfill \\ \vdots \hfill & \vdots \hfill & \vdots \hfill & \vdots \hfill \\ 0 \hfill & 0 \hfill & \cdots \hfill & {C_{iK} } \hfill \\ \end{array} } \right]$$

Because the bootstrap process disturbs the order of data, in order to calculate the training set of the subclassifier $D_{i}$, each column in the matrix $R_{i}$ needs to be reordered according to the original feature set. The rotation matrix obtained after reordering is denoted as $R_{i}^{\alpha } \in {\mathbb{R}}^{N \times n}$. For subclassifier $D_{i}$, the training set after the rotation transformation is $X^{\prime } = XR_{i}^{\alpha }$.

The fourth step, in the classification phase, the new sample $x$ also needs to conduct rotation transformation, and the new sample after the rotation transformation is $x^{\prime } = xR_{i}^{\alpha }$. We let $d_{ij} \left( {xR_{i}^{\alpha } } \right)$ be the subclassifier $D_{i}$ to determine the probability that the sample $x$ belongs to classes 1 or 2, and the credibility of assigning the sample to a certain class is:

9 $$\mu_{j} \left( x \right) = \frac{1}{L}\sum {d_{ij} \left( {xR_{i}^{\alpha } } \right)} \, \quad j = 1,2$$

Sample $x$ judges the category to which it belongs with maximum credibility, where $L$ represents the number of subclassifiers, and 1 or 2 indicate the sample belonging to positive or negative.

In this study, we used SVM as the subclassifier for the Rotation Forest integrated algorithm.

### Construct and evaluate model

To further improve the performance of predicting carbonylation and non-carbonylation sites, the Rotation Forest integrated algorithm was utilized by using a majority voting strategy to integrate the predictive results of subclassifiers. The performance of CarSite-II was evaluated using the following six measurements: Sensitivity (Sn), Specificity (Sp), Accuracy (Acc), Matthew’s correlation coefficient (MCC), geometric mean (G-mean) and the area under the receiver operating characteristic curves (AUC), which were defined as follows:

10 $$Sn = 1 - \frac{{N_{ - }^{ + } }}{{N^{ + } }}$$

11 $$Sp = 1 - \frac{{N_{ + }^{ - } }}{{N^{ - } }}$$

12 $$Acc = 1 - \frac{{N_{ - }^{ + } + N_{ + }^{ - } }}{{N^{ + } + N^{ - } }}$$

13 $$MCC = \frac{{1 - \left( {\frac{{N_{ - }^{ + } + N_{ + }^{ - } }}{{N^{ + } + N^{ - } }}} \right)}}{{\sqrt {\left( {1 + \frac{{N_{ + }^{ - } - N_{ - }^{ + } }}{{N^{ + } }}} \right)\left( {1 + \frac{{N_{ - }^{ + } - N_{ + }^{ - } }}{{N^{ - } }}} \right)} }}$$

14 $$G - mean = \sqrt {Sn \times Sp}$$

15 $$AUC = \frac{{\sum\nolimits_{i = 1}^{{N^{ + } }} {\sum\nolimits_{j = 1}^{{N^{ - } }} {u\left( {f_{i}^{ + } ,f_{j}^{ - } } \right)} } }}{{N^{ + } \times N^{ - } }}$$

in which,

$$u\left( {\overline{x},\tilde{x}} \right) = \left\{ {\begin{array}{*{20}l} {1,} \hfill & {\overline{x} > \tilde{x}} \hfill \\ {0,} \hfill & {otherwise} \hfill \\ \end{array} } \right.$$

Here $N^{ + }$ represented the size of carbonylation sequences, while $N_{ - }^{ + }$ indicated the total number of carbonylation sequences which were incorrectly predicted as non-carbonylation sequences; $N^{ - }$ represented the number of non-carbonylation sequences, while $N_{ + }^{\_}$ was the total number of non-carbonylation sequences, which were incorrectly predicted as carbonylation sequences, $f_{i}^{ + }$ was the score of the *i*th positive sample, and $f_{j}^{ - }$ was the score of the *j*th negative sample.

## Supplementary Information

**Additional file 1:** SupTable (SubTable1.1–SubTable1.4. The predictive performance of K/P/R/T carbonylation sites by 10-fold cross validation).

**Additional file 2:** SubTable 2. The Wilcoxon signde rank of the K/P/R/T carbonylation sites.

**Additional file 3:** SubTable 3. Web-Server Guide.

## Data Availability

All data generated during this study and the algorithm available to download and run locally in either http://47.100.136.41:8081/dataSet or its additional files.

## Abbreviations

SMOTE-KSU: K-means similarity-based undersampling and the synthetic minority oversampling technique
MCC: Matthew’s correlation coefficient
DR: Distance-based residue
SVM: Support vector machine
K: Lys
R: Arginine
P: Proline
T: Threonine
