# Supplementary material for: CarSite-II: an integrated classification algorithm for identifying carbonylated sites based on K-means similarity-based undersampling and synthetic minority oversampling techniques
Source: BMC Bioinformatics. 2021 Apr 26;22:216. doi: 10.1186/s12859-021-04134-3 (PMC8077735; doi:10.1186/s12859-021-04134-3)
Supplement: Supplementary file 1 — Additional file 1: SupTable (SubTable1.1–SubTable1.4. The predictive performance of K/P/R/T carbonylation sites by 10-fold cross validation). [file 12859_2021_4134_MOESM1_ESM.docx]

**SupTable1.1 The predictive performance of K carbonylation sites by 10-fold cross validation**

| **The number of K** | **features** | **Sn (%)** | **Sp (%)** | **Acc (%)** | **Mcc** | **AUC** | **G-mean** |
| --- | --- | --- | --- | --- | --- | --- | --- |
| 300 | DR(1) | 75.20 | 72.68 | 72.79 | 0.2129 | 0.8150 | 0.7393 |
|  | DR(2) | 72.28 | 71.24 | 71.28 | 0.1906 | 0.7963 | 0.7176 |
|  | DR(3) | 73.75 | 72.28 | 72.33 | 0.2038 | 0.8002 | 0.7301 |
| 310 | DR(1) | 77.53 | 73.14 | 73.31 | 0.2250 | 0.8177 | 0.7530 |
|  | DR(2) | 73.04 | 72.01 | 72.05 | 0.1988 | 0.7971 | 0.7252 |
|  | DR(3) | 72.99 | 72.36 | 72.37 | 0.2007 | 0.8047 | 0.7267 |
| 320 | DR(1) | 75.98 | 73.09 | 73.21 | 0.2183 | 0.8197 | 0.7452 |
|  | DR(2) | 74.39 | 71.69 | 71.81 | 0.2033 | 0.7990 | 0.7303 |
|  | DR(3) | 75.25 | 72.67 | 72.78 | 0.2128 | 0.8075 | 0.7395 |
| 330 | DR(1) | 76.74 | 73.27 | 73.40 | 0.2224 | 0.8219 | 0.7498 |
|  | DR(2) | 72.70 | 72.12 | 72.14 | 0.1977 | 0.8037 | 0.7241 |
|  | DR(3) | 73.35 | 72.66 | 72.69 | 0.2042 | 0.8089 | 0.7300 |
| 340 | DR(1) | 75.85 | 73.25 | 73.35 | 0.2183 | 0.8211 | 0.7454 |
|  | DR(2) | 76.61 | 71.72 | 71.93 | 0.2123 | 0.8065 | 0.7412 |
|  | DR(3) | 73.34 | 72.70 | 72.74 | 0.2048 | 0.8060 | 0.7302 |
| 350 | DR(1) | 74.13 | 71.94 | 72.04 | 0.2043 | 0.8048 | 0.7303 |
|  | DR(2) | 73.99 | 72.10 | 72.19 | 0.2036 | 0.8034 | 0.7304 |
|  | DR(3) | 71.48 | 72.86 | 72.80 | 0.1977 | 0.8051 | 0.7217 |
| 360 | DR(1) | 76.53 | 73.83 | 73.93 | 0.2254 | 0.8219 | 0.7517 |
|  | DR(2) | 74.00 | 72.08 | 72.16 | 0.2031 | 0.8029 | 0.7303 |
|  | DR(3) | 73.27 | 72.98 | 72.99 | 0.2062 | 0.8104 | 0.7312 |
| 370 | DR(1) | 77.54 | 73.54 | 73.69 | 0.2278 | 0.8267 | 0.7551 |
|  | DR(2) | 71.67 | 72.24 | 72.20 | 0.1940 | 0.8005 | 0.7195 |
|  | DR(3) | 73.06 | 72.44 | 72.47 | 0.2021 | 0.8062 | 0.7275 |
| 380 | DR(1) | 75.21 | 73.07 | 73.16 | 0.2147 | 0.8220 | 0.7413 |
|  | DR(2) | 74.41 | 71.85 | 71.97 | 0.2044 | 0.8001 | 0.7312 |
|  | DR(3) | 72.29 | 73.09 | 73.05 | 0.2026 | 0.8069 | 0.7269 |
| 390 | DR(1) | 74.36 | 73.38 | 73.42 | 0.2135 | 0.8140 | 0.7387 |
|  | DR(2) | 73.25 | 71.73 | 71.78 | 0.1977 | 0.8004 | 0.7249 |
|  | DR(3) | 74.43 | 72.62 | 72.71 | 0.2086 | 0.8050 | 0.7352 |
| 400 | DR(1) | 77.40 | 73.37 | 73.54 | 0.2255 | 0.8250 | 0.7536 |
|  | DR(2) | 74.62 | 71.89 | 72.02 | 0.2058 | 0.8034 | 0.7324 |
|  | **DR(3)** | **77.48** | **73.72** | **73.87** | **0.2287** | **0.8263** | **0.7558** |

**SupTable1.2 The predictive performance of P carbonylation sites by 10-fold cross validation**

| **The number of K** | **features** | **Sn (%)** | **Sp (%)** | **Acc (%)** | **Mcc** | **AUC** | **G-mean** |
| --- | --- | --- | --- | --- | --- | --- | --- |
| 300 | DR(1) | 92.10 | 87.00 | 87.07 | 0.2675 | 0.9467 | 0.8951 |
|  | DR(2) | 94.31 | 86.19 | 86.30 | 0.2652 | 0.9436 | 0.9016 |
|  | DR(3) | 92.54 | 86.55 | 86.63 | 0.2635 | 0.9433 | 0.8949 |
| 310 | DR(1) | 92.56 | 87.29 | 87.36 | 0.2726 | 0.9478 | 0.8989 |
|  | DR(2) | 95.39 | 86.38 | 86.51 | 0.2690 | 0.9447 | 0.9077 |
|  | DR(3) | 91.70 | 87.01 | 87.08 | 0.2632 | 0.9454 | 0.8932 |
| 320 | DR(1) | 91.54 | 87.49 | 87.55 | 0.2695 | 0.9483 | 0.8949 |
|  | DR(2) | 94.87 | 86.79 | 86.91 | 0.2722 | 0.9447 | 0.9074 |
|  | DR(3) | 93.78 | 87.21 | 87.29 | 0.2725 | 0.9488 | 0.9044 |
| 330 | DR(1) | 93.02 | 87.65 | 87.72 | 0.2766 | 0.9490 | 0.9030 |
|  | DR(2) | 94.44 | 87.14 | 87.24 | 0.2746 | 0.9502 | 0.9072 |
|  | DR(3) | 93.55 | 87.38 | 87.46 | 0.2736 | 0.9498 | 0.9041 |
| 340 | DR(1) | 94.60 | 87.85 | 87.94 | 0.2863 | 0.9506 | 0.9116 |
|  | DR(2) | 94.26 | 87.68 | 87.77 | 0.2836 | 0.9526 | 0.9091 |
|  | DR(3) | 93.54 | 87.69 | 87.76 | 0.2802 | 0.9508 | 0.9057 |
| 350 | DR(1) | 94.47 | 88.07 | 88.15 | 0.2857 | 0.9535 | 0.9121 |
|  | DR(2) | 94.51 | 87.58 | 87.68 | 0.2823 | 0.9532 | 0.9098 |
|  | DR(3) | 91.63 | 88.24 | 88.31 | 0.2819 | 0.9515 | 0.8992 |
| 360 | DR(1) | 94.14 | 88.62 | 88.70 | 0.2912 | 0.9534 | 0.9134 |
|  | DR(2) | 95.08 | 87.84 | 87.95 | 0.2868 | 0.9545 | 0.9139 |
|  | DR(3) | 91.82 | 88.38 | 88.44 | 0.2834 | 0.9520 | 0.9008 |
| 370 | DR(1) | 94.19 | 88.64 | 88.72 | 0.2936 | 0.9560 | 0.9137 |
|  | DR(2) | 95.39 | 88.46 | 88.56 | 0.2964 | 0.9583 | 0.9186 |
|  | DR(3) | 93.43 | 88.56 | 88.64 | 0.2926 | 0.9548 | 0.9096 |
| 380 | DR(1) | 95.53 | 88.94 | 89.03 | 0.3001 | 0.9570 | 0.9218 |
|  | DR(2) | 95.90 | 88.49 | 88.60 | 0.2981 | 0.9583 | 0.9212 |
|  | DR(3) | 94.41 | 88.86 | 88.94 | 0.2979 | 0.9575 | 0.9159 |
| 390 | DR(1) | 94.36 | 89.10 | 89.17 | 0.3022 | 0.9579 | 0.9169 |
|  | DR(2) | 97.20 | 88.61 | 88.72 | 0.3048 | 0.9601 | 0.9281 |
|  | DR(3) | 94.90 | 89.16 | 89.23 | 0.3038 | 0.9589 | 0.9199 |
| 400 | DR(1) | 95.14 | 89.29 | 89.37 | 0.3076 | 0.9580 | 0.9217 |
|  | **DR(2)** | **97.95** | **89.16** | **89.28** | **0.3125** | **0.9634** | **0.9345** |
|  | DR(3) | 94.48 | 89.34 | 89.42 | 0.3069 | 0.9603 | 0.9187 |

**SupTable1.3 The predictive performance of R carbonylation sites by 10-fold cross validation**

| **The number of K** | **features** | **Sn (%)** | **Sp (%)** | **Acc (%)** | **Mcc** | **AUC** | **G-mean** |
| --- | --- | --- | --- | --- | --- | --- | --- |
| 300 | DR(1) | 88.59 | 83.21 | 83.31 | 0.2439 | 0.9209 | 0.8586 |
|  | DR(2) | 87.95 | 82.89 | 82.99 | 0.2392 | 0.9124 | 0.8538 |
|  | DR(3) | 85.95 | 83.09 | 83.15 | 0.2332 | 0.9091 | 0.8451 |
| 310 | DR(1) | 89.46 | 83.66 | 83.75 | 0.2498 | 0.9234 | 0.8651 |
|  | DR(2) | 89.91 | 83.34 | 83.45 | 0.2488 | 0.9167 | 0.8656 |
|  | DR(3) | 88.11 | 84.06 | 84.12 | 0.2467 | 0.9252 | 0.8606 |
| 320 | DR(1) | 89.58 | 83.90 | 84.00 | 0.2528 | 0.9257 | 0.8669 |
|  | DR(2) | 89.49 | 83.55 | 83.66 | 0.2498 | 0.9181 | 0.8647 |
|  | DR(3) | 89.33 | 83.93 | 84.02 | 0.2525 | 0.9225 | 0.8659 |
| 330 | DR(1) | 90.66 | 84.27 | 84.37 | 0.2586 | 0.9287 | 0.8741 |
|  | DR(2) | 89.03 | 83.43 | 83.53 | 0.2466 | 0.9175 | 0.8618 |
|  | DR(3) | 89.69 | 84.56 | 84.64 | 0.2576 | 0.9252 | 0.8709 |
| 340 | DR(1) | 89.19 | 84.51 | 84.60 | 0.2568 | 0.9290 | 0.8682 |
|  | DR(2) | 90.48 | 83.69 | 83.81 | 0.2540 | 0.9210 | 0.8702 |
|  | DR(3) | 88.50 | 84.32 | 84.38 | 0.2527 | 0.9271 | 0.8638 |
| 350 | DR(1) | 89.35 | 84.76 | 84.84 | 0.2599 | 0.9310 | 0.8702 |
|  | DR(2) | 87.91 | 83.73 | 83.82 | 0.2467 | 0.9207 | 0.8579 |
|  | DR(3) | 89.08 | 84.97 | 85.03 | 0.2590 | 0.9317 | 0.8700 |
| 360 | DR(1) | 88.53 | 85.01 | 85.10 | 0.2594 | 0.9308 | 0.8675 |
|  | DR(2) | 91.94 | 84.07 | 84.20 | 0.2595 | 0.9259 | 0.8792 |
|  | DR(3) | 87.00 | 84.94 | 85.00 | 0.2542 | 0.9250 | 0.8596 |
| 370 | DR(1) | 89.84 | 85.37 | 85.46 | 0.2678 | 0.9347 | 0.8758 |
|  | DR(2) | 91.16 | 84.47 | 84.59 | 0.2624 | 0.9283 | 0.8775 |
|  | DR(3) | 87.78 | 85.06 | 85.11 | 0.2568 | 0.9312 | 0.8641 |
| 380 | DR(1) | 90.84 | 85.44 | 85.53 | 0.2715 | 0.9372 | 0.8810 |
|  | DR(2) | 91.79 | 84.77 | 84.88 | 0.2671 | 0.9321 | 0.8821 |
|  | DR(3) | 87.99 | 85.40 | 85.45 | 0.2624 | 0.9332 | 0.8669 |
| 390 | DR(1) | 90.54 | 85.61 | 85.69 | 0.2720 | 0.9383 | 0.8804 |
|  | DR(2) | 92.55 | 84.88 | 84.99 | 0.2676 | 0.9324 | 0.8863 |
|  | DR(3) | 88.72 | 85.58 | 85.64 | 0.2656 | 0.9361 | 0.8714 |
| 400 | **DR(1)** | **90.24** | **86.17** | **86.26** | **0.2787** | **0.9364** | **0.8818** |
|  | DR(2) | 93.68 | 85.03 | 85.18 | 0.2779 | 0.9344 | 0.8925 |
|  | DR(3) | 87.37 | 85.61 | 85.64 | 0.2627 | 0.9342 | 0.8649 |

**SupTable1.4 The predictive performance of T carbonylation sites by 10-fold cross validation**

| **The number of K** | **features** | **Sn (%)** | **Sp (%)** | **Acc (%)** | **Mcc** | **AUC** | **G-mean** |
| --- | --- | --- | --- | --- | --- | --- | --- |
| 300 | DR(1) | 90.14 | 82.90 | 83.01 | 0.2309 | 0.9222 | 0.8644 |
|  | DR(2) | 88.99 | 83.07 | 83.16 | 0.2308 | 0.9233 | 0.8598 |
|  | DR(3) | 89.54 | 82.99 | 83.09 | 0.2300 | 0.9190 | 0.8620 |
| 310 | DR(1) | 92.86 | 83.40 | 83.53 | 0.2434 | 0.9261 | 0.8800 |
|  | DR(2) | 89.19 | 83.42 | 83.51 | 0.2334 | 0.9283 | 0.8626 |
|  | DR(3) | 91.36 | 84.61 | 84.72 | 0.2506 | 0.9290 | 0.8792 |
| 320 | DR(1) | 91.20 | 83.72 | 83.83 | 0.2413 | 0.9285 | 0.8738 |
|  | DR(2) | 88.95 | 83.49 | 83.59 | 0.2326 | 0.9279 | 0.8618 |
|  | DR(3) | 91.93 | 85.22 | 85.31 | 0.2581 | 0.9335 | 0.8851 |
| 330 | DR(1) | 89.99 | 84.40 | 84.48 | 0.2433 | 0.9319 | 0.8715 |
|  | DR(2) | 90.04 | 84.14 | 84.23 | 0.2423 | 0.9322 | 0.8704 |
|  | DR(3) | 90.61 | 85.37 | 85.45 | 0.2542 | 0.9355 | 0.8795 |
| 340 | DR(1) | 91.48 | 84.79 | 84.90 | 0.2523 | 0.9331 | 0.8807 |
|  | DR(2) | 91.62 | 84.67 | 84.78 | 0.2521 | 0.9331 | 0.8808 |
|  | DR(3) | 89.95 | 85.65 | 85.73 | 0.2575 | 0.9327 | 0.8777 |
| 350 | DR(1) | 91.97 | 85.17 | 85.27 | 0.2578 | 0.9355 | 0.8850 |
|  | DR(2) | 91.94 | 84.81 | 84.92 | 0.2540 | 0.9378 | 0.8830 |
|  | DR(3) | 91.91 | 85.85 | 85.93 | 0.2646 | 0.9392 | 0.8883 |
| 360 | DR(1) | 90.62 | 85.52 | 85.60 | 0.2566 | 0.9387 | 0.8803 |
|  | DR(2) | 92.48 | 84.94 | 85.06 | 0.2538 | 0.9402 | 0.8863 |
|  | DR(3) | 92.42 | 86.20 | 86.30 | 0.2696 | 0.9422 | 0.8926 |
| 370 | DR(1) | 91.96 | 85.60 | 85.70 | 0.2613 | 0.9375 | 0.8872 |
|  | DR(2) | 92.10 | 85.61 | 85.72 | 0.2634 | 0.9404 | 0.8880 |
|  | DR(3) | 91.82 | 86.18 | 86.27 | 0.2680 | 0.9406 | 0.8896 |
| 380 | DR(1) | 92.19 | 85.74 | 85.85 | 0.2649 | 0.9377 | 0.8891 |
|  | DR(2) | 92.45 | 85.71 | 85.82 | 0.2654 | 0.9410 | 0.8902 |
|  | DR(3) | 92.31 | 86.40 | 86.49 | 0.2713 | 0.9449 | 0.8931 |
| 390 | DR(1) | 92.23 | 86.17 | 86.26 | 0.2696 | 0.9398 | 0.8915 |
|  | DR(2) | 95.57 | 86.12 | 86.26 | 0.2768 | 0.9456 | 0.9072 |
|  | DR(3) | 93.14 | 86.63 | 86.74 | 0.2798 | 0.9469 | 0.8983 |
| 400 | DR(1) | 93.28 | 86.23 | 86.34 | 0.2704 | 0.9423 | 0.8969 |
|  | DR(2) | 93.90 | 86.35 | 86.47 | 0.2758 | 0.9469 | 0.9005 |
|  | **DR(3)** | **93.92** | **86.87** | **87.56** | **0.2814** | **0.9513** | **0.9033** |
