# Supplementary material for: CarSite-II: an integrated classification algorithm for identifying carbonylated sites based on K-means similarity-based undersampling and synthetic minority oversampling techniques
Source: BMC Bioinformatics. 2021 Apr 26;22:216. doi: 10.1186/s12859-021-04134-3 (PMC8077735; doi:10.1186/s12859-021-04134-3)
Supplement: Supplementary file 2 — Additional file 2: SubTable 2. The Wilcoxon signde rank of the K/P/R/T carbonylation sites. [file 12859_2021_4134_MOESM2_ESM.docx]

Web-Server Guide

Since user-friendly and publicly accessible web-servers represent the future direction for developing practically more useful predictors, here we have provided a web-server for the method presented in this paper at http://47.100.136.41:8081.

For the convenience of the vast majority of experimental scientists, let us give a step-by-step guide on how to use the CarSite-II web server to get their desired results without the need to follow the complicated mathematic equations that were presented just for the integrity in developing the predictor. The detailed steps are as follows：

**Step 1.** Open the web-server at http://47.100.136.41:8081 and you will see the homepage of CarSite-II web-server as shown in the following:


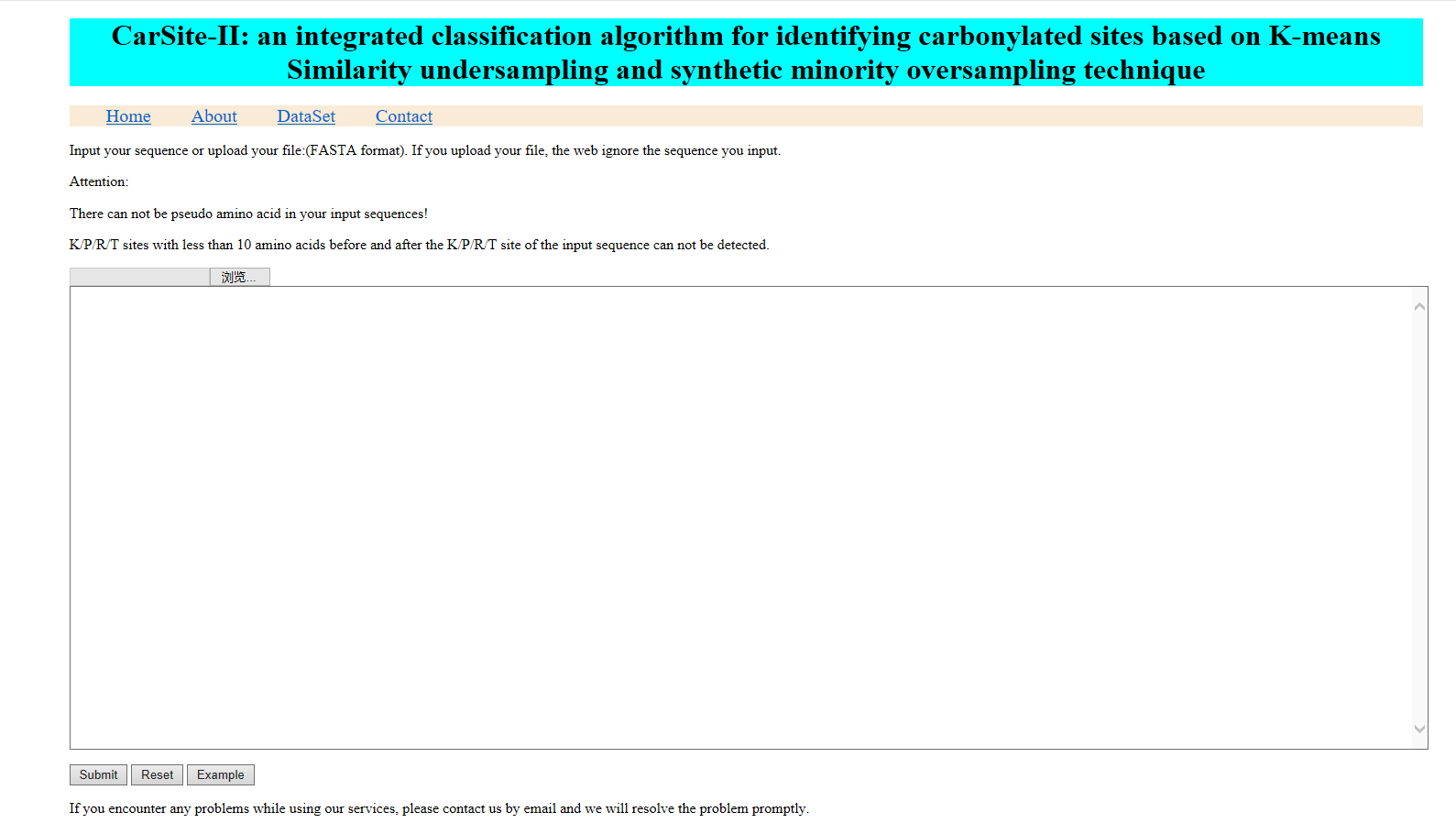


**Step 2.** Paste or upload the query protein sequence into the field below：


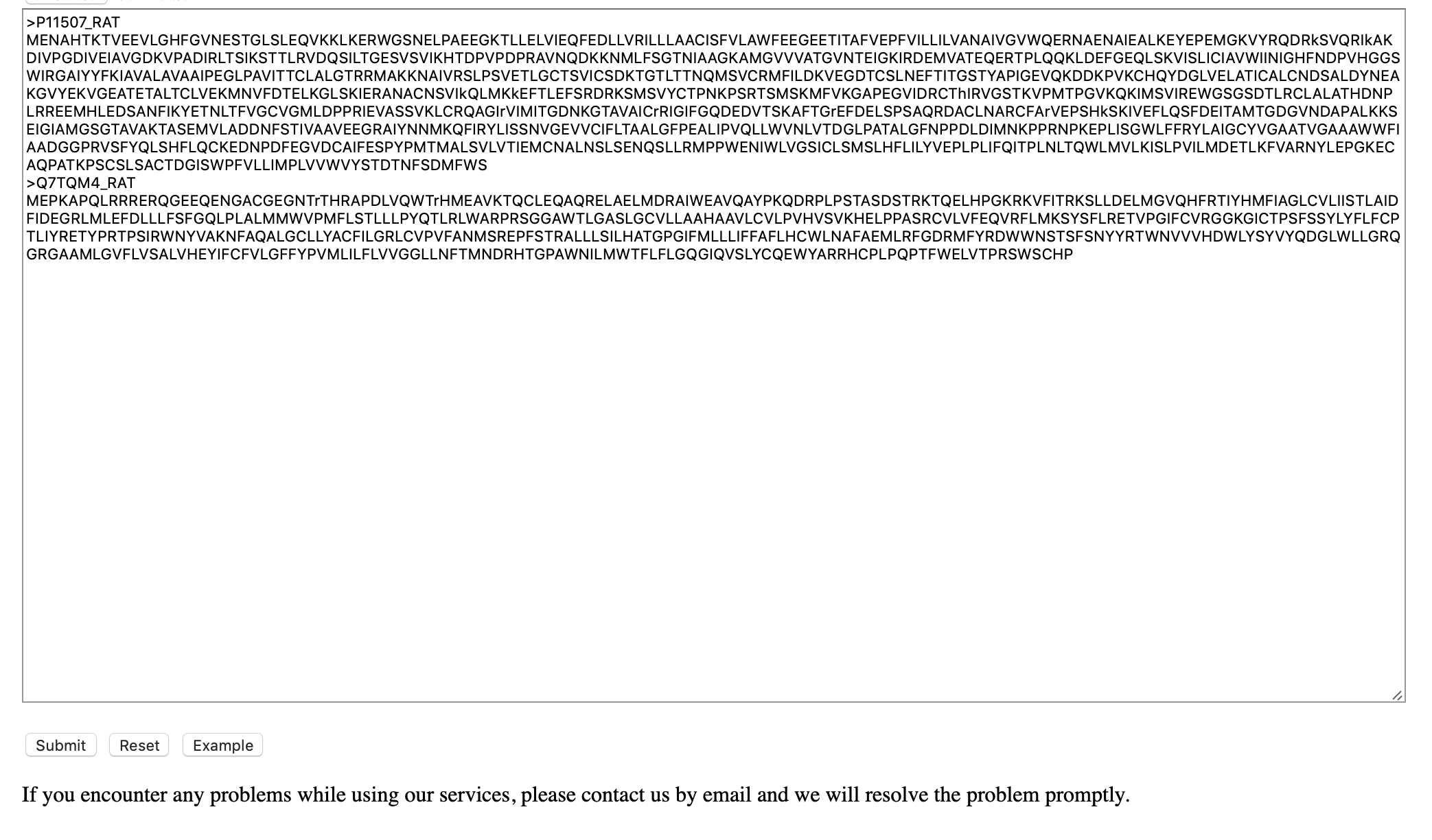


The input sequence should be in **FASTA** format. The first line of FASTA format is the name of protein and its format is a symbol of ’ > ’ followed by a series of characters. The other lines are the sequences of protein. Example sequences in FASTA format can be found by clicking on the Example button.

**Step 3.** Click on the Submit button, you will see the predictive results as follows:


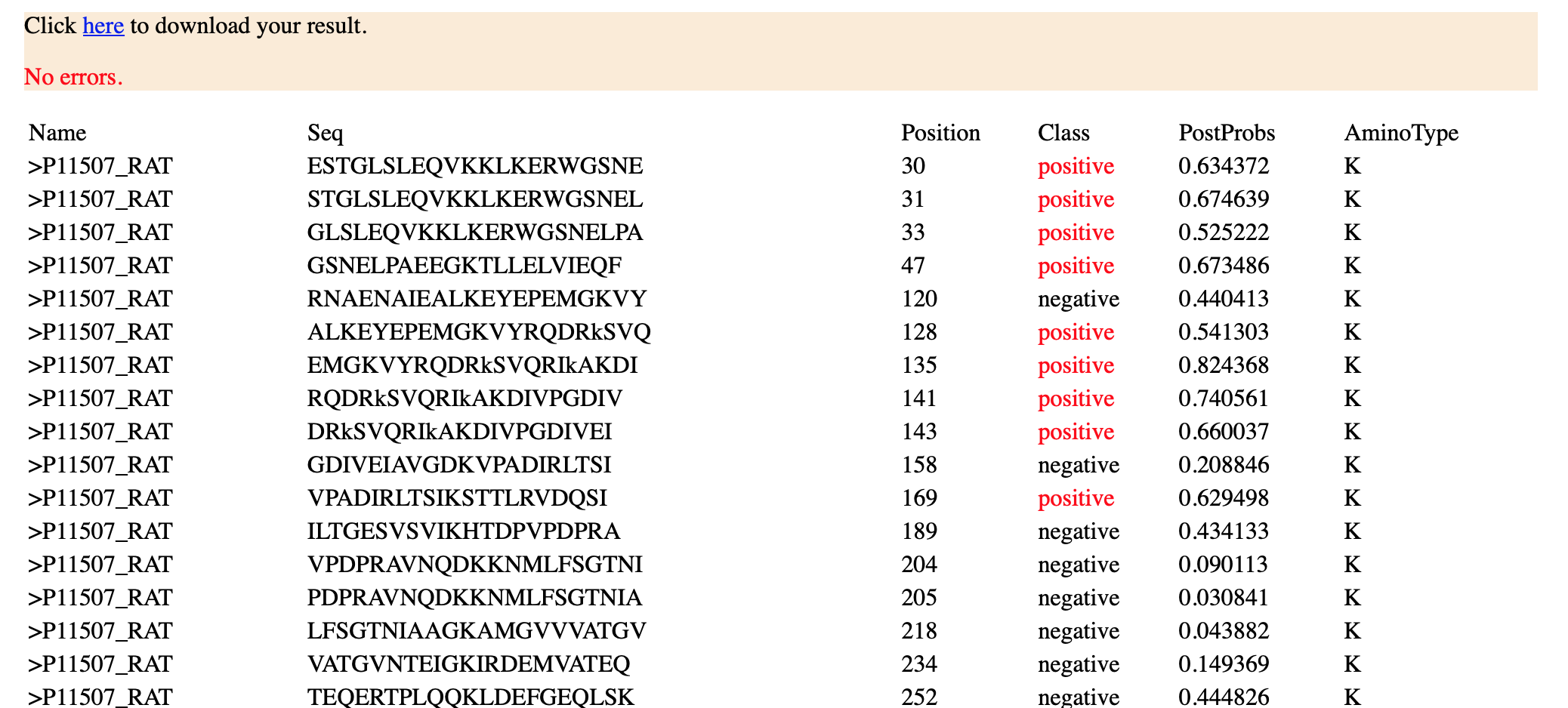


**Step 4.** Click here to download your result as follows:


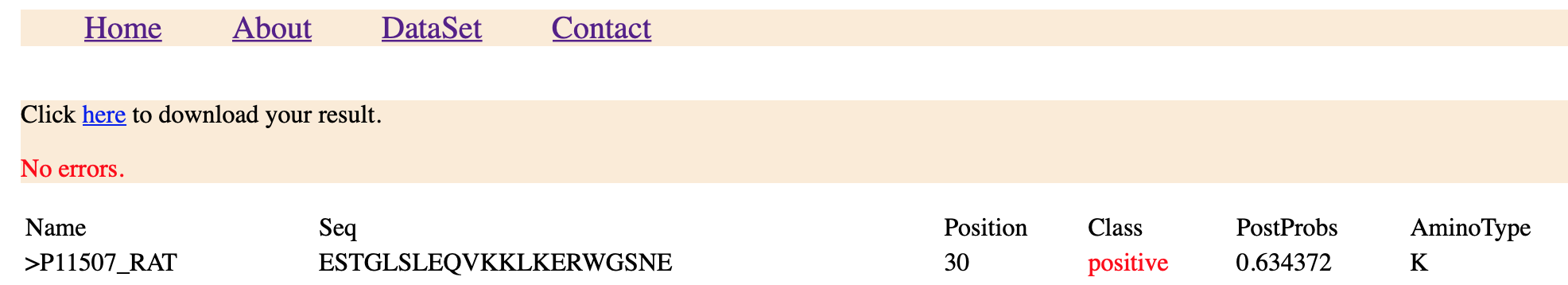


**Attention:**

(1)A. When inputting a sequence without target sites, the following error message will be reported:


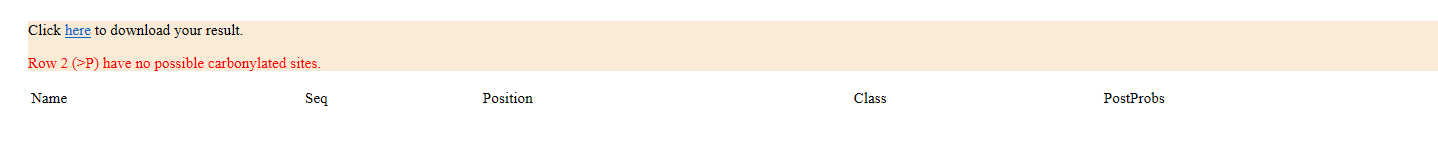
Row 2 (>xx) have no possible carbonylated sites. The concrete output showed in the following:


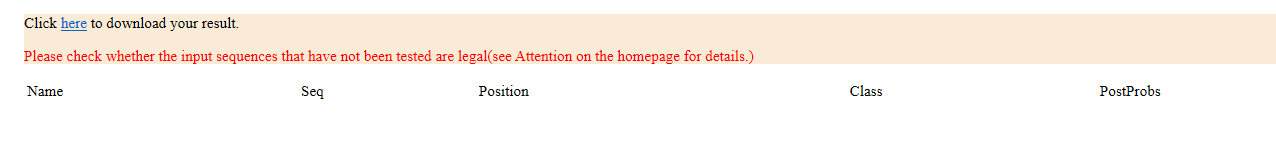
B. When inputting a sequence with less than 10 amino acids before and after target sites of the input sequence, the following error message will be reported: Please check whether the input sequences that have not been tested are legal (see Attention on the homepage for details.) The concrete output showed in the following:

(2) When the input sequences have both sequences without a target site and a normal sequence, for untested sequences, the following error message will be reported: Please check whether the input sequences that have not been tested are legal (see Attention on the homepage for details.) The concrete output showed in the following:


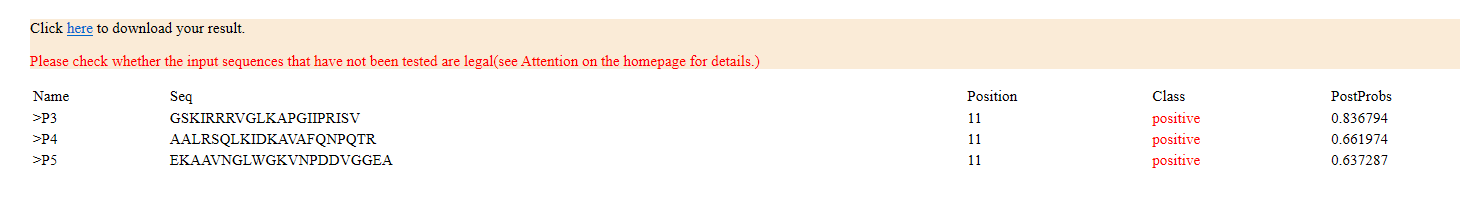


(3)Input your sequence or upload your file: (FASTA format). If you upload your file, the web ignore the sequence you input.

(4)There can not be pseudo amino acid in your input sequences!

(5)K/P/R/T sites with less than 10 amino acids before and after the K/P/R/T site of the input sequence can not be detected.

If you encounter any problems while using our services, please contact us by email([xrliu@xmu.edu.cn](mailto:xrliu@xmu.edu.cn) or [zydpdl123@163.com](mailto:zydpdl123@163.com) or [linjianyuan@stu.xmu.edu.cn](mailto:linjianyuan@stu.xmu.edu.cn)) and we will resolve the problem as soon as possible.
